# Supplementary material for: Structural modeling and analysis of fuel cell: a graph-theoretic approach
Source: PeerJ Comput Sci. 2023 Aug 28;9:e1510. doi: 10.7717/peerj-cs.1510 (PMC10495936; doi:10.7717/peerj-cs.1510)
Supplement: Supplemental Information 1 — The program is based on Laplace expansion. [file peerj-cs-09-1510-s001.pdf]

Structural Modeling and Analysis of Fuel Cell: A Graph-Theoretic Approach

Code of Computer Program for Solving NXN Matrix

For the calculation of permanent function of NXN matrix is given in this appendix and value of N can be upto 50. The program is based upon Laplace expansion. Suppose B=(b_{ij}) is an NXN matrix then its determinant is given by:

$$|B| = \sum_{j=1}^{N} b_{ij} C_{ij}$$

$$and\ C_{ij} = (-1)^{i+j} M_{ij}$$

Where M_{ij} is the i, j minor matrix of B, that is, the determinant of the (n–1) × (n–1) matrix that results from deleting the i-th row and the j-th column of B. For the calculation of permanent function, the cofactor C_{ij} is defined as:

$$C_{ij} = M_{ij}$$

Due to this all negative signs in matrix expansion are converted in to positive signs

```
Variables Used int total Column Number of columns and rows in the matrix double
matrix[50][50]
The matrix input by User int z[50]
Variable Array used for calculation of perof2
Functions Used
double per(int) Recursive function that calculates the value of permanent
double perof2(); Calculates the permanent of last 2 elements
*************************************************************************/
int totalColumn;
double matrix[50][50];
int z[50];
double per(int);
double perof2();
/*************************************************************************
Local Variables for main function
int i Temporary integer variable
int j Temporary integer variable
double k Double variable that stores the value returned by per()
*************************************************************************/
void main()
{
int i,j;
double k;
clrscr();
printf("Enter the size of the matrix:");
scanf("%d",&totalColumn);
/*Input from user the elements of Matrix that is n*n, i.e., totalColumn*totalColumn*/
```

```
printf("\n Enter the elements of matrix:\n");
for (i = 0;i<=totalColumn-1;i++)
{
for(j = 0;j<=totalColumn-1;j++)
{
printf(" Element [%d][%d]: ",i+1,j+1);
scanf("%lf",&matrix[i][j]);
}
}
printf("\n\n\n\n\nPress any key to continue….");
getch();
clrscr();
/*Show to user the matrix formed or inputted*/
printf("The matrix is: \n\n");
printf("\n\t");
for(i=0;i<=totalColumn-1;i++)
{
for(j=0;j<=totalColumn-1;j++)
{
printf("%1f ",matrix[i][j]);
}
printf("\n\t");
}
k=per(totalColumn);
printf("\n\nThe final Value is %lf",k);
getch();
}
/***********************************************************************
This algorithm works as follows... It calculates the values in a row number, i.e., firstly it will
call the value of the permanent for first element in the first row, then adds the permanent of
second element of the first row and the process continues...
z keeps the check for per2, what is to be calculated for per2. per will make the values for all z
elements '1' except those 2 elements whose per2 has to be calculated.
Variables used in per
int i Temporary integer variable
double res Double variable for storing the value returned from per()
double res2 Double variable for storing the value returned from perof2()
double sum Variable for calculating the sum
***********************************************************************/
double per(int n)
{
int i;
double res, res2, sum=0;
if((n-2)!=0)
{
int c=0;
for(i=1;i<=totalColumn;i++)
{
if(z[i]==0)
{
```

```
z[i]=1;
z[c]=0;
c=i;
res=per(n-1);
res=(double)(res*matrix[totalColumn-n][i-1]);
sum=sum+res;
}
}
z[c]=0;
}
else
{
res2=perof2();
return(res2);
}
return(sum);
}
/**************************************************************************
This function calculates the value of the matrix 2*2. The 2*2 matrix is defined by the array z[].
Variables used in perof2
int i,j Temporary integer variable
int n,m Temporary integer variable
int flag Contains value 0 or 1, acts as Boolean
double res Double variable for storing the value calculated by multiplication
**************************************************************************/
double perof2()
{
int i,j,flag=0,n,m;
double res;
for(i=1;i<=totalColumn;i++)
{
if(z[i]==0)
{
if(flag==0)
{
n=i;
flag=1;
}
else
m=i;
}
}
res=(double)((matrix[totalColumn-2][n-1]*matrix[totalColumn-1][m-
1])+(matrix[totalColumn-2][m-1]*matrix[totalColumn-1][n-1]));
return res;
}
```
